# Evaluation of Heat and pH Treatments on Degradation of Ceftiofur in Whole Milk

**DOI:** 10.3389/fvets.2020.00288

**Published:** 2020-05-22

**Authors:** Adriana Garzon, Pramod Pandey, Lisa Tell, Sharif S. Aly, Robert Poppenga, Richard Pereira

**Affiliations:** ^1^Department of Population Health and Reproduction, School of Veterinary Medicine, University of California, Davis, Davis, CA, United States; ^2^Department of Medicine and Epidemiology, School of Veterinary Medicine, University of California, Davis, Davis, CA, United States; ^3^Veterinary Medicine Teaching and Research Center, University of California, Davis, Tulare, CA, United States; ^4^California Animal Health and Food Safety Laboratory, Davis, CA, United States

**Keywords:** antibiotics, β-lactams, drug residue, inactivation, waste milk

## Abstract

Waste milk feeding practices have been implicated as a potential source for disseminating antimicrobial resistant bacteria among animals and the environment. Two interventions that have shown potential for degrading antimicrobial drugs in milk are heat and pH treatment. The aim of this study was to evaluate the effect of heat and pH treatments on the degradation of ceftiofur and ceftiofur free acid equivalents in milk at concentrations previously found in waste milk on dairy farms by spiking saleable pasteurized whole milk with ceftiofur sodium. Three heat treatments of ceftiofur sodium spiked milk were evaluated for their ability to degrade ceftiofur: 63°C for 30 min (LTLT), 72°C for 15 s (HTST) and 92°C for 20 min (HTLT). Two pH treatments of ceftiofur sodium spiked milk were evaluated: pH 4.0 (LpH) and pH 10 (HpH). Control samples spiked with ceftiofur sodium were kept at room temperature and samples collected at corresponding times for heat and pH treatments. Four treatment replicates were performed for each treatment group. Ceftiofur was quantified in milk samples using liquid chromatography mass spectrometry (LC-MS/MS) and ceftiofur free acid equivalents (CFAE) were measured using high-performance liquid chromatography (HPLC). HTLT resulted in a degradation of 35.24% of the initial concentration of ceftiofur. Ceftiofur degradation did not differ between control and the remaining two heat treatment groups (LTLT and HTST). HpH resulted in degradation of the 95.72 and 96.28% of the initial concentration of ceftiofur and CFAE, respectively. No significant changes in degradation of ceftiofur or CFAE were observed for control or LpH treatments. In conclusion, our study results were that alkalinizing milk to pH 10 and heating milk to 92°C for 20 min degraded ceftiofur and CFAE in spiked simulated waste milk demonstrated promising potential as treatment options for degrading ceftiofur and CFAE in waste milk, and further research is needed to evaluate the viability for implementation of these treatments in dairy farms.

## Introduction

Antimicrobials are undoubtedly one of the most important tools for preventing and treating diseases. Decreasing the rate of selection for drug resistance is of importance to both human and veterinary medicine. Non-saleable milk, also known as waste milk, is milk withheld due to pharmaceutical residues from lactating cows receiving drugs for therapeutic reasons. To reduce production losses due to waste milk, 30.6% of dairy farms in the U.S. feed waste milk to preweaned calves (1). Feeding calves waste milk has also been associated with antibiotic residues violations (2, 3). Slaughter withdrawal intervals recommendations for veal calves fed colostrum from cows receiving antibiotics during the dry period have been estimated (4). The disposal of waste milk with pharmaceutical residues can be laborious and costly to dairy farmers and could still represent a potential source for selection of resistance in the environment (5). There is therefore a need for approaches that would allow the sustainable use of waste milk without the selection of antimicrobial resistance or other unwanted outcomes.

A study by Pereira et al. (6), evaluated the impact of feeding waste milk spiked with residual concentrations of ampicillin, penicillin, ceftiofur, and tetracycline, according to the most prevalent drugs previously identified in waste milk on New York dairies (7). By the end of the trial, calves fed with milk spiked with antimicrobials had significantly higher proportions of *E. coli* resistant to one of six different antimicrobials, as well as multidrug resistant (MDR) *E. coli* (resistant to 3 or more drugs) compared to control calves fed milk without added antimicrobials (71% MDR treatment vs. 13% MDR control, *P* <0.0001). Decreasing drug residues in milk could avoid the deleterious impacts of feeding waste milk on selection of drug resistance.

Degradation of β-lactam antibiotics in aqueous solutions is influenced by temperature and pH (8, 9). Ceftiofur beta-lactam is unstable in aqueous base (pH 10.0) and acid (pH 3.0) solutions (8). Acidification of milk to a pH between 4.0 to 4.5 and fed to preweaned calves is a practice that has become common in recent years in the US, with the objective of lowering the milk pH to a point where it is unsuitable for bacterial growth and survival without undesirable health side effects on calves (10). The impact of acidification of waste milk on drug residue degradation is currently unknown. Heat treatment of waste milk to reduce bacterial counts could potentially be an option for antimicrobial degradation. Roca et al. (11) reported degradation of 41.2% of cephapirin (a first-generation cephalosporin) in milk, after samples were heat at 63°C for 30 min, and further degradation of different beta-lactam drugs occurred when samples were exposed to higher temperatures (100°C). The aim of this study was to evaluate the effect of heat and pH treatments on the concentration of ceftiofur and ceftiofur free acid equivalents (CFAE) in milk, added at concentrations previously observed in waste milk on dairy farms.

## Materials and Methods

### Spiked Milk Samples

Saleable pasteurized homogenized whole milk (3.25% fat content) was spiked using stock solutions of ceftiofur, as previously described (6). Briefly, 60 mg of ceftiofur sodium powder (93.6% purity, Sigma-Aldrich, St. Louis, MO) was diluted in 93.6 ml of distilled water (Millipore Corp., Bedford, MA) with 0.96% of dimethyl sulfoxide (Cell Signaling Technology, Danvers, MA, USA) added to increase the solubility of ceftiofur, to a stock concentration of 600 μg/ml, which was used to spike a volume of 3 l of milk, to a final concentration of 200 ppb for heat treatment trials and 400 ppb for pH trials. Stock solutions were stored in individual vials at −80°C until used. Concentrations of ceftiofur targeted in milk batches were based on previously reported concentrations of ceftiofur in waste milk on dairy farms in the US (7, 12).

A total of four repetitions with new milk batches were conducted for each heat treatment and pH assay. This number of repetitions was based on reported references for heat and pH stability of antimicrobials, where we estimate an 80.5% statistical power to identify a significant difference between samples after treatment when compared to the control group (α = 0.05, standard deviation = 0.22, difference to detect = 0.18) (8, 11, 13).

### Heat Treatment

Three heat treatments were evaluated, where two temperature and time combinations were based on pasteurization treatments used for waste milk on dairy farms: low temperature—long time (**LTLT**), where samples were heated to 63°C (145°F) and held at that temperature for 30 min; high temperature—short time (**HTST**), where samples were heated to 72°C (161°F) for 15 s; and high temperature—long time (**HTLT**), where samples were heated to 92°C (197.6°F) and held at that temperature for 20 min. A control group was maintained at room temperature, and samples from this group were collected at the corresponding times for the three heat treatment samples.

The same initial milk batch spiked with ceftiofur was divided in four aliquots and used for each heat treatment group to reduce between treatment group variations for each repetition. Collected samples were stored at −80°C until drug quantification. Four replicates were performed for each treatment group. Outline of heat treatment procedure is displayed in Supplemental Figure 1.

### pH Treatment

Two pH treatments were evaluated: low pH group (**LpH**), prepared by adding diluted formic acid to milk and gently stirring until a pH of 4.0 was achieved; and high pH group (**HpH**), prepared by adding sodium hydroxide to milk samples and gently stirring until a pH of 10.0 was achieved. The pH was measured using a pH meter (basic pH meter 840087, Sper Scientific ltd., Scottsdale, AZ). A control milk group (pH ~6.5–6.7) kept at room temperature was used as a control sample, and samples from the control group were collected at the same time points as samples for the pH treatment groups.

Similar to heat treatment protocol, the same initial milk batch was divided in three aliquots after spiking with ceftiofur and used for each pH treatment and control group. Samples collected were stored at −80°C until drug quantification. Four replicates were performed for each treatment group. All milk treatment groups were gently stirred before samples were collected at each time point, as well as every 6 h after beginning of testing. Outline of pH treatment procedure is displayed in Supplemental Figure 2.

### Chromatographic Analysis

Ceftiofur was quantified in samples using liquid chromatography mass spectrometry (LC-MS/MS) at the California Animal Health & Food Safety toxicology laboratory (Davis, CA). This approach only quantified ceftiofur, and not desfuroylceftiofur. Sample analysis was performed using a LC-MS/MS method described in the Food and Drug Administration (FDA) LIB# 4443 (14). The limit of quantification (LOQ) of the assay was 100 ppb of ceftiofur in milk. Samples below the limit of detection after treatment were analyzed using 10 ppb of ceftiofur as final concentration.

Concentrations of ceftiofur free acid equivalents (CFAE) were measured using high-performance liquid chromatography (HPLC). CFAE was quantified only for samples from the high pH treatment group, due to significant ceftiofur degradation in the high pH trial. The method has been described in a previous study (15). Briefly, dithioerythritol was used to cleave any macromolecules bound to desfuroylceftiofur in milk and to convert the parent drug and metabolites to desfurylceftiofur. The sample was then run through a C18 solid phase extraction (SPE) column (Thermo Scientific, Rockwood, TN, USA) and derivatized with iodoacetamide to create desfuroylceftiofur acetamide. After elution from the C18 SPE column, further clean-up was done on a strong cation exchange (SCX) SPE (Agilent Technologies, Santa Clara, CA, USA). The HPLC analysis was done isocratically (mobile phase was 7% acetonitrile, 1% acetic acid, with 90 mg heptane sulphonic acid/liter, and pH = 4.0) on a Nova-Pak C18, 4μm, 3.9 X 150 mm (Waters Corporation, Milford, MA, USA) with UV detection at 240 nm. The standard curve was made in milk with a range from 0.01 to 1.0 μg/ml. Quality control samples were spiked to obtain a final concentration of 200 ppb for heat treatment trials and 400 ppb of Ceftiofur for the pH trials, with average recovery rate of 94%.

### Statistical Analysis

Assumption of normality for ceftiofur and CFAE concentration from pH treatment trials was tested using Shapiro-Wilk W test, and assumption of homogeneity of variance was tested using Levene's test using JMP. If assumptions were maintained, analysis was conducted using JMP Pro 14 (SAS Institute, Cary, NC). To evaluate the effect of pH treatment over time on the degradation of ceftiofur and CFAE, multivariate mixed models were fitted to the data using the GLIMMIX procedure of SAS. Two models were generated where the dependent variables were ceftiofur and CFAE. Independent variables offered to the model were treatment (e.g., control, LpH, and HpH), sampling time points and the interaction between treatment and time points. The effect of individual sample identifier as well as trial number was controlled in all the models as a random effect. Because samples from HpH resulted in multiple ceftiofur concentrations in milk below the limit of detection (10 ppb), a more conservative approach was used to evaluate the data where samples with a ceftiofur concentration below the 10 ppb detection level, were labeled as having a ceftiofur concentration of 10 ppb. Tukey-Krammer pairwise comparison between all different treatment groups and time points was conducted. When either Shapiro-Wilk W test or Levene's test was rejected, the non-parametric Dunn All Pairs for joint ranks test in JMP was used to evaluate if there was a significant difference in the ceftiofur concentration in milk between pH treatment groups and control samples for each time point using pairwise approach. The Dunn All Pairs for joint ranks test was chosen because it has been shown to be a better choice because it has been shown to be a more powerful test for detecting differences between extreme treatments, and because joint ranking procedure have been shown to have slightly higher power than the pairwise ranking, reducing the risk of type 2 errors (16).

Assumption of normality for ceftiofur concentration from temperature treatment trials was tested using Shapiro-Wilk W test, and assumption of homogeneity of variance was tested using Levene's test using JMP. To evaluate the effect of heat treatment over time on the degradation of ceftiofur, multivariate mixed models were fitted to the data using the GLIMMIX procedure of SAS. Independent variables offered to the model were treatment (e.g., control, HTLT, LTLT, and HTST), sampling time points and the interaction between treatment and time points. When either Shapiro-Wilk W test or Levene's test was rejected, the non-parametric Dunn All Pairs for joint ranks test in JMP was used to evaluate if there was a significant difference in the ceftiofur concentration in milk between temperature treatment groups and control samples for each time point using pairwise approach. A *P* ≤ 0.05 was considered statistically significant for analysis conducted in this study.

## Results

### Heat Treatment Group

**Figure 1:**
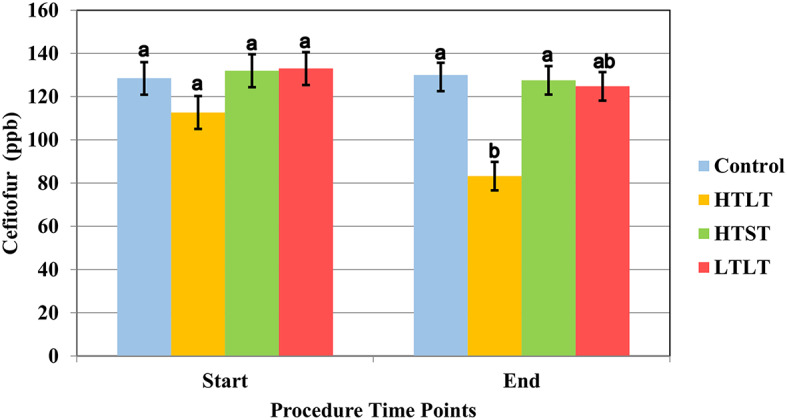
Least square mean of ceftiofur (LSM ± SD) upon target temperature (start) was reached and at the end of each heat treatment protocol for milk samples heated at 63°C for 30 min (**LTLT**), 72°C for 15 s (**HTST**) and 92°C for 20 min (**HTLT**) and control. Letter reflect the results for Dunn All Pairs for joint ranks non-parametric test, and different letter indicate a significant difference between treatment group within each time point.

The results of the heat treatment assay are displayed in Figure 1. The least square means (**LSM**) for the initial concentration of ceftiofur for the heat treatment was 128.5 ppb (95% confidence interval 121.07–135.96). Data for ceftiofur concentration for heat treatment rejected the Shapiro-Wilk W test, and the Dunn All Pairs for joint ranks test was used for this analysis.

Control sample collected from pool of milk following spiking and mixing of milk at room temperature, prior to any heat treatment, did not significantly differ from control samples collected at timepoints 15 s, 20 min, and 30 min (Supplemental Table 1). There was a significant degradation of ceftiofur when samples were heated at 92°C and held to that temperature for 20 min (HTLT) compared to the control group (*P* <0.016) (Supplemental Table 1), with the final LSM for ceftiofur at 83.22 ppb (CI 95% 76.62–89.81). The degradation of ceftiofur in milk did not significantly differ between the control, HTST and LTLT, with LSM observed at 129.9 (CI 95% 124.29–135.68), 127.56 ppb (CI 95% 120.96–134.15) and 124.78 ppb (CI 95% 118.18–131.37), respectively (Figure 1 and Supplemental Table 1).

### pH Treatment Group

Ceftiofur concentration for treatment group HpH was below detection levels for 8 of 12 samples at timepoint “0,” which was collected immediately after adding sodium hydroxide to milk samples and gently stirring until a pH of 10.0 was achieved. For HpH group, all samples collected at timepoints 12 h and 24 h were below the detection limits (10 ppb for ceftiofur). Data for ceftiofur concentration for pH treatment rejected the Shapiro-Wilk W test, and the Dunn All Pairs for joint ranks test was used for this analysis.

**Figure 2:**
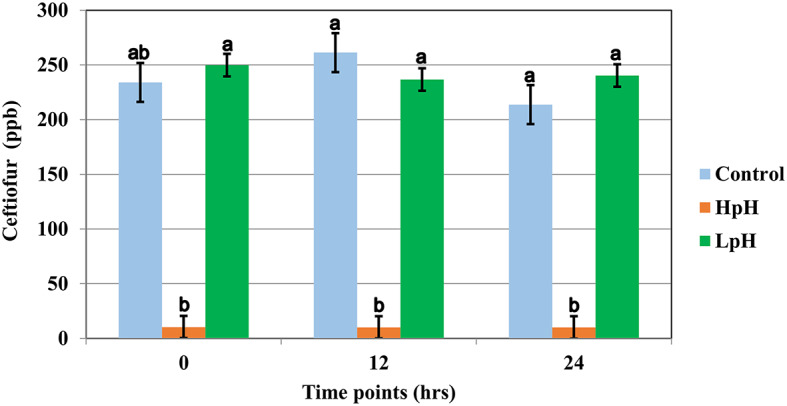
Least square mean of ceftiofur (LSM ± SD) following spiking milk (Ct), upon target pH, and 12 and 24 h after target pH was reached for milk samples reaching a pH = 10 (**HpH**), milk samples reaching a pH = 4.0 (**LpH**) and control sample. Letter reflect the results for Dunn All Pairs for joint ranks non-parametric test, and different letter indicate a significant difference between treatment group within each time point.

The results of the pH treatment assay are displayed in Figure 2. The LSM for the initial concentration of ceftiofur for pH treatment was 234 ppb (CI 95% 216.19–251.80). The LSM concentration of ceftiofur was 213.75 ppb (CI 95% 195.94–231.55) for normal pH and 240.33 ppb (CI 95% 230.04–250.61) for low pH but declined to 10 (CI 95% −0.28–20.28 ppb) ppb immediately after sodium hydroxide was added and pH 10 was achieved, resulting in a significant degradation of ceftiofur when compared to the control group (*P* <0.0001) (Figure 2 and Supplemental Table 2).

Control sample collected from pool of milk following spiking and mixing of milk at room temperature, prior to any pH treatment, did not significantly differ from control samples collected at timepoints 12 h and 24 h (Supplemental Table 2).

### Quantification of Ceftiofur Free Acid Equivalents

**Figure 3:**
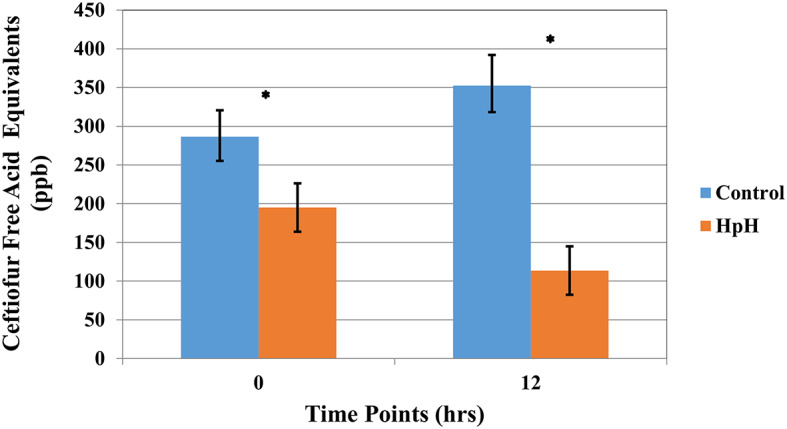
Least square mean of ceftiofur free acid equivalents (CFAE) (LSM ± SD) following spiking milk (Ct), upon target pH, and 12 h after target pH was reached for milk samples reaching a pH = 10 (**HpH**) and control sample. Asterisk represents time points where a significant difference was observed between HpH and control for that same time point.

The results of the high pH treatment assay on the concentration of CFAE in ceftiofur spiked whole milk are shown in Figure 3. Neither normal variance nor equal variance assumptions for the use of a liner regression model were rejected for the CFAE dataset. The mean initial concentration of CFAE was 286.5 ppb (CI 95% 252.40–320.59). The concentration of CFAE in samples decreased to a mean of 113.58 ppb (CI 95% 82.1–144.84) after milk was alkalized to pH 10, resulting in a significant degradation of ceftiofur when compared to the control group (*P* <0.0001) (Figure 3 and Supplemental Table 3).

## Discussion

Heating ceftiofur spiked milk at 92°C for 20 min resulted in a significant reduction in ceftiofur and CFAE concentrations when compared to the control treatment. Similar to our findings, Zorraquino et al. (17) evaluated heat treatment of five cephalosporin drugs, not including ceftiofur, and observed an inactivation of over 90% of cephlosporin drugs tested when milk samples were heat treated at 92°C for 20 min, and between 6 and 18% degradation when heat treated at 60°C for 30 min. A difference compared to our study is that their study did not measure drug concentrations using chromatographic methods but instead a bioassay based on the inhibition of *Geobacillus stearothermophilus* var. *calidolactis*. A potential concern with heating milk at 92°C is the possible effect on nutrient content. Higher temperatures have been shown to decrease the percentage of soluble whey proteins in milk due to denaturalization (18). Our results support that further research should be conducted to evaluate the viability of introducing an approach that uses temperatures higher than those traditionally used in the dairy industry.

No significant degradation of either ceftiofur or CFAE was observed using the HTST or LTLT treatments when compared to the control group, indicating that time as well as heating temperature are critical factor for effective ceftiofur degradation. HTST and LTLT are common practices for pasteurization of waste milk fed to calves with the goal of lowering bacterial contamination (19). A study by Li et al. (20) evaluated the effect of temperature on the degradation of ceftiofur in aqueous solutions with or without addition of recycled water derived from a beef farm. Samples were incubated at 15, 25, 35, and 45°C. Ceftiofur hydrolysis rate in deionized water without wastewater increased from 0.1 to 5.4 ×10^−3^ h^−1^ as temperature increased from 10 to 45°C, which represented a hydrolysis rate increase of 3.8 times by each 10°C increased in temperature. A difference in our study was the effect of all other components in milk that can results in a different degradation dynamic then that observed in water. Half-lives of cephalosporins other than of ceftiofur in milk, have been shown to be between 32 and 90 min at 70°C, and 40 to 127 min at 60°C (11). Horton et al. (21) reported that for complete degradation of cefquinome, a fourth generation cephalosporin, milk required a heat treatment at 50°C for more than 72 h, resulting in a 86% degradation after 48 h of incubation (t_½_ = 30.9 h). Our results indicated that hydrolysis of cephalosporins at 63°C and 72°C may require longer time than standard pasteurization protocols currently being used by the dairy industry.

Treatment of ceftiofur spiked milk using a pH of 10 resulted in a significant and prompt degradation of ceftiofur and CFAE, although the latter occurred at a slower pace. A study by Horton et al. (21) observed similar results, with increasing milk pH to 10.0 resulting in a reduction of cefquinome concentration, a fourth-generation cephalosporin, below the limit of detection (<125 μg/kg) within 8 h. A potential concern with alkalinizing milk is the potential effects on nutrients, as well as safety as a food product for calves. Increasing milk pH to 10.0 has been demonstrated to decreased casein micellar size and milk turbidity, that did not return to the initial levels after milk pH was adjusted to a normal milk pH, indicating a permanent alteration of casein micelles (22). This permanent change in caseins structure may affect the nutritional value of waste milk. Another possible aspect that may influence the applicability of alkalization of milk is palatability as well as the effect on bacteria growth, which to our knowledge, has not been estimated. Further studies are needed to evaluate the effect of alkaline treatment on milk quality as well as approaches to adjust final pH and supplementation of additional nutrients in the milk before feeding calves.

Acidification of milk to a pH of 4.0 did not result in a significant degradation of ceftiofur. Other studies have indicated that acid-catalyzed hydrolysis had a negligible effect on degradation of other β-lactams (23, 24). In a study by Mitchel et al. (24), hydrolysis rates of three β-lactam antibiotics were evaluated using acetate and borate buffers at pH 4.0–9.0, incubating samples at 25, 50, and 60°C. The calculated half-lives of cefalotin (first-generation cephalosporin), cefoxitin (second-generation cephalosporin) and ampicillin at pH 4.0 and 25°C were 5.2, 9.3, and 3 days, respectively. First and second generation cephalosporins may differ in degradation pathways to third generation cephalosporins (e.g., ceftiofur), which could affect hydrolysis rate and half-lives of the components. Gilbertson et al. (23) observed similar results to our study when evaluated ceftiofur degradation on acetate (pH 5.0), phosphate (pH 7.0), and borate (pH 9.0) buffers incubated at 22 and 47°C.

In the Gilbertson et al. (23) study the reported half-lives of ceftiofur at 22°C were 100.3, 8 and 4.2 d at pH 5.0, 7.0 and 9.0, respectively. Even though, half-lives of antibiotics between both studies were considerably different (23, 24), they were still longer than the time evaluated in our study, which explain pH 4.0 did not increase ceftiofur degradation in milk samples. One difference between both studies and ours is that they evaluated antibiotic degradation using buffers solutions instead of milk, which may influence the degradation rate of antibiotics. Acidification of waste milk is a preservation method used to inhibit bacterial growth and survival without affects its nutritional value (25). Lowering milk pH to 4.0 using formic acid has shown to reduce coliform and aerobic bacterial growth in milk replacers (26) and raw bulk tank milk (27), as well as decrease diarrhea episodes in calves in compare with pasteurized and untreated waste milk (28). Acidified milk is fed by 1.7% of farms in the United States (29), and if successful, may represent a cost-effective strategy to treat antimicrobial residues in milk. Furthermore, our study provides novel information to clarify that waste milk acidification as a bacteria inhibition process cannot be assumed to have an effect on degradation of ceftiofur residues.

Desfuroylceftiofur is the main metabolite product of ceftiofur hydrolysis (30). Free desfuroylceftiofur is an active metabolite with the intact cephalosporin part of the molecule responsible for biological activity. Desfuroylceftiofur is the marker residue for ceftiofur, with a tolerance level in milk of 0.1 ppm. The marker residue is the residue whose concentration is in a known relationship to the concentration of total residue in edible tissue (31). An approach to measure both free desfuroylceftiofur and conjugated ceftiofur is to quantify the ceftiofur-free acid equivalents (CFAE) (32). In our study, given the significant degradation of ceftiofur observed when milk pH was increased to 10.0, CFAE concentrations were also evaluated to determine if ceftiofur was just being converted to another microbiologically active metabolite.

## Conclusion

Heat and pH and treatments might be alternative cost effective on-farm strategies that could increase the degradation of antimicrobials on waste milk. Adding sodium hydroxide to ceftiofur spiked milk until pH 10 was achieved increased the degradation of ceftiofur and CFAE in milk. Heating ceftiofur spiked milk to 92°C for 20 min also decreased ceftiofur concentrations in spiked milk samples but to a much lesser extent. Further studies to evaluate the possibility of using these approaches on farms are needed, including palatability, adjusting final treatment products to allow safe consumption of milk by calves, and evaluating if these alternative methods reduce the potential for antimicrobial resistance when feeding antibiotic contaminated waste milk to calves.

## Data Availability Statement

The datasets generated for this study are available on request to the corresponding author.

## Author Contributions

RPe, PP, and SA contributed conception and design of the study. RPo, PP, RPe, and LT contributed to conducting the experiment and analytical testing of samples. RPe and AG performed the statistical analysis. RPe and AG wrote the first draft of the manuscript. All authors contributed to manuscript revision, read, and approved the submitted version.

## Conflict of Interest

The authors declare that the research was conducted in the absence of any commercial or financial relationships that could be construed as a potential conflict of interest.
