# Supplementary material for: Evaluation of Heat and pH Treatments on Degradation of Ceftiofur in Whole Milk
Source: Front Vet Sci. 2020 May 22;7:288. doi: 10.3389/fvets.2020.00288 (PMC7256783; doi:10.3389/fvets.2020.00288)
Supplement: Supplementary file 1 [file Data_Sheet_1.pdf]

SUPPLEMENTAL FIGURES

Supplemental Figure 1. Outline of assay for treatment of milk samples spiked with ceftiofur using heat treatment. For each temperature, four batch replicates were conducted, collecting samples for each time point in triplicate (except for control samples). Black filled cells indicate time points when samples were collected, and number within each cell indicates number of samples collected for each batch. **Ct**: milk collected following spiking and mixing of milk at room temperature, prior to any heat treatment. *sample was collected once milk reached desired treatment temperature. **LTLT:** 145º F (63ºC) for 30 min; **HTST:** 161°F (72°C) for 15 seconds; **HTLT:** 197.6º F (92ºC) for 20 min.

	**Sampling Time Points**				
**Treatment Groups**	**Ct**	**0***	**15 sec**	**20 min**	**30 min**
LTLT	3	1			3
HTST		1	3		
HTLT		1		3	
Control			1	1	1

Supplemental Figure 2. Outline of assay for treatment of milk spiked with ceftiofur using pH. For each pH treatment group, four batch replicates were conducted, collecting samples for each time point in triplicate (except for control samples). Black filled cells indicate time points when samples were collected, and number within each cell indicates number of samples collected for each batch. **Ct**: milk collected following spiking and mixing of milk at room temperature, prior to any pH treatment. * sample was collected once milk reached treatment desired pH. **HpH:** pH = 10.0; **LpH:** pH = 4.0.

	**Sampling Time Points**			
**Treatment Groups**	**Ct**	**0***	**12 hrs**	**24 hrs**
HpH	1	3	3	3
LpH		3	3	3
Control			1	1

Ct: milk collected following spiking and mixing of milk at room temperature, prior to any pH treatment. * sample was collected once milk reached treatment desired pH. **HpH:** pH = 10.0; **LpH:** pH = 4.0.
