# Supplementary material for: Evaluation of Heat and pH Treatments on Degradation of Ceftiofur in Whole Milk
Source: Front Vet Sci. 2020 May 22;7:288. doi: 10.3389/fvets.2020.00288 (PMC7256783; doi:10.3389/fvets.2020.00288)
Supplement: Supplementary file 2 [file Data_Sheet_2.PDF]

SUPPLEMENTAL TABLES

Supplemental Table 1. Output from the non-parametric Dunn All Pairs for joint ranks test comparing ceftiofur degradation between heat treatment group and control samples for each time point. *P* values in bold indicate pairwise comparison with statistical significance.

Time Points/Tx Groups[1]	SMD[2]	SE[3]	Z[4]	*P* value[5]
**Time Point = Start**				
**HTLT vs LTLT**	-9.8	4.3	2.3	0.14
**HTLT vs HTST**	-9.3	4.3	-2.1	0.19
**LTLT vs HTST**	0.2	4.3	0.04	1
**HTLT vs Control[6]**	-7.5	3.5	-2.1	0.20
**HTST vs Control[6]**	1.7	3.5	0.5	1
**LTLT vs Control[6]**	2.2	3.5	0.6	1
**Time Point = End**				
**HTLT vs LTLT**	-13.8	4.9	-2.8	0.07
**HTLT vs HTST**	-18.0	4.9	-3.6	**0.004**
**LTLT vs HTST**	-4.0	4.9	-0.8	1
**HTLT vs Control_HTLT[7]**	-21.3	7.0	-3.0	**0.03**
**HTST vs Control_HTST[8]**	-1.8	7.0	-0.3	1
**LTLT vs Control_LTLT[9]**	-10.0	7.0	-1.4	1

1. Time point comparisons for each treatment group; the first treatment group served as the reference for each pairwise analysis. 2. Score mean difference; 3. Standard error of the SMD; 4. Z-value for Wilcoxon's test; 5. *P* value for the Dunn All Pairs for joint ranks test; 6. Control sample from pool of milk collected following spiking and mixing of milk at room temperature, prior to any heat treatment. 7. Control samples at environmental temperature collected at the same time HTLT sample was collected at time point "End"; 8. Control samples at environmental temperature collected at the same time HTST sample was collected at time point "End"; 9. Control samples at environmental temperature collected at the same time LTLT sample was collected at time point "End".

Supplemental Table 2. Output from the non-parametric Dunn All Pairs for joint ranks test comparing ceftiofur degradation between pH treatment groups and control samples for each time point. *P* values in bold indicate pairwise comparison with statistical significance.

Time Points/Tx Groups[1]	SMD[2]	SE[3]	Z[4]	*P* value[5]
**Time Point = 0***				
**HpH vs LpH**	-15.0	3.3	-4.5	**<0.0001**
**HpH vs Control[6]**	-10.6	4.6	-2.2	0.07
**LpH vs Control[6]**	4.1	4.6	0.9	1
**Time Point = 12 hours**				
**HpH vs LpH**	-12.3	3.2	-3.8	**0.0004**
**HpH vs Control[7]**	-18.6	4.5	-4.0	**0.0001**
**LpH vs Control[7]**	-6.2	4.5	-1.3	0.52
**Time Point = 24 hours**				
**HpH vs LpH**	-14.1	3.2	-4.4	**<0.0001**
**HpH vs Control[7]**	-13.1	4.5	-2.9	**0.012**
**LpH vs Control[7]**	0.8	4.5	0.2	1

1. Time point comparisons for each treatment group; the first treatment group served as the reference for each pairwise analysis. 2. Score mean difference; 3. Standard error of the SMD; 4. Z-value for Wilcoxon's test; 5. *P* value for the Dunn All Pairs for joint ranks test; 6. Control sample from pool of milk collected following spiking and mixing of milk at room temperature, prior to any pH treatment. 7. Control samples maintained at environmental temperature and collected respectively at 12 hours and 24 hours. *Sample was collected once milk reached treatment desired pH.
