# Supplementary material for: Evaluation of Heat and pH Treatments on Degradation of Ceftiofur in Whole Milk
Source: Front Vet Sci. 2020 May 22;7:288. doi: 10.3389/fvets.2020.00288 (PMC7256783; doi:10.3389/fvets.2020.00288)
Supplement: Supplementary file 3 [file Table_3.docx]

**Supplemental Table 3.** Least square mean difference of ceftiofur free acid equivalents (CFAE) (LSM ± SD) for comparing time points and treatment groups, upon target pH (0 h), and 12 hours after target pH was reached for milk samples reaching a pH=10 (**HpH**) and control sample, using Tukey-Krammer pairwise analysis (Figure 5).

| Factor* | LSM Diff | SE | *P* value** |
| --- | --- | --- | --- |
| HpH vs Control (0 h) | -91.5 | 20.2 | 0.0018 |
| HpH vs Control (12 h) | -238.7 | 22.7 | **<.0001** |

* First combination factors displayed serve as reference value.

** *P* value for multiple comparisons using Tukey-Krammer for different treatment and time point combinations.
